# Melatonin antagonizes lipopolysaccharide-induced pulpal fibroblast responses

**DOI:** 10.1186/s12903-020-1055-3

**Published:** 2020-03-29

**Authors:** Nutthapong Kantrong, Piyabhorn Jit-Armart, Uthaiwan Arayatrakoollikit

**Affiliations:** 1 Department of Restorative Dentistry, Faculty of Dentistry, Khon Kaen University, Mittraphap road, Nai Mueang, Mueang, Khon Kaen, 40002 Thailand; 2 Oral Biology Research Unit, Faculty of Dentistry, Khon Kaen University, Khon Kaen, Thailand; 3 Research Group of Chronic Inflammatory Oral Diseases and Systemic Diseases Associated with Oral Health, Faculty of Dentistry, Khon Kaen University, Khon Kaen, Thailand; 4 Wanon-Niwat Hospital, Wanon-Niwat, Sakon Nakhon, Thailand

**Keywords:** Melatonin, Melatonin receptor, Dental pulp, Pulp cells, Pulpal fibroblasts, Pulpal inflammation

## Abstract

**Background:**

Pulpal inflammation is known to be mediated by multiple signaling pathways. However, whether melatonin plays regulatory roles in pulpal inflammation remains unclear. This study aimed at elucidating an in situ expression of melatonin and its receptors in human pulpal tissues, and the contribution of melatonin on the antagonism of lipopolysaccharide (LPS)-infected pulpal fibroblasts.

**Methods:**

Melatonin expression in pulpal tissues harvested from healthy teeth was investigated by immunohistochemical staining. Its receptors, melatonin receptor 1 (MT1) and melatonin receptor 2 (MT2), were also immunostained in pulpal tissues isolated from healthy teeth and inflamed teeth diagnosed with irreversible pulpitis. Morphometric analysis was subsequently performed. After LPS infection of cultured pulpal fibroblasts, cyclo-oxygenase (COX) and interleukin-1 β (IL-1 β) transcripts were examined by using reverse transcription-polymerase chain reaction (RT-PCR). Analysis of mRNA expression was performed to investigate an antagonism of LPS stimulation by melatonin via COX and IL-1 β induction. Mann-Whitney U test and One-way ANOVA were used for statistical analysis to determine a significance level.

**Results:**

Melatonin was expressed in healthy pulpal tissue within the odontoblastic zone, cell-rich zone, and in the pulpal connective tissue. Furthermore, in health, strong MT1 and MT2 expression was distributed similarly in all 3 pulpal zones. In contrast, during disease, expression of MT2 was reduced in inflamed pulpal tissues (*P*-value< 0.001), but not MT1 (*P*-value = 0.559). Co-culturing of melatonin with LPS resulted in the reduction of COX-2 and IL-1 β expression in primary pulpal fibroblasts, indicating that melatonin may play an antagonistic role to LPS infection in pulpal fibroblasts.

**Conclusions:**

Human dental pulp abundantly expressed melatonin and its receptors MT1 and MT2 in the odontoblastic layers and pulpal connective tissue layers. Melatonin exerted antagonistic activity against LPS-mediated COX-2 and IL-1 β induction in pulpal fibroblasts, suggesting its therapeutic potential for pulpal inflammation and a possible role of pulpal melatonin in an immunomodulation via functional melatonin receptors expressed in dental pulp.

## Background

Pulpal inflammation is an outcome of complex host-bacteria interactions that involves multiple intracellular machineries in tooth pulp. Early stage of pulpal injury caused by bacterial stimuli is mediated via innate immune sensors i.e. Toll-like receptor (TLR), particularly TLR4 which recognizes lipopolysaccharide (LPS), a potent immunostimulatory component derived from gram-negative bacterial cell walls [1, 2]. Recent study has shown that LPS-mediated inflammation of the pulp leads to an upregulation of an array of inflammatory cytokines and mediators including interleukin-1 β (IL-1 β), tumor necrosis factor- α (TNF- α) and antimicrobial peptides such as human β-defensins, chemokine CCL20, [1], thereby ensuring rapid innate immune defense in response to microbial challenge in dental pulp.

In deep dental caries, the chance of chronic exposure of pulp cells to polymicrobial infections is dramatically increased due to the abundance of cariogenic bacterial species found in carious dentin [3]. Such microorganisms may cause potential dangers to the dental pulp upon their direct engagement with innate immune receptors. An unavoidable microbial burden that reaches the dental pulp via dentinal tubules triggers not only an increased expression profile of inflammatory mediators, but also an elevated amount of host protective protein such as transforming growth factor (TGF)- β 1 [4]. Strikingly, the interaction of TGF- β 1 with odontoblasts results in dampened pulpal immune response via suppression of TLR4 [5]. Interestingly, LPS stimulation of human pulpal cells leads to the synthesis of cyclooxygenase (COX) enzymes [6], underscoring the implication of TLR4 in modulating pulpal inflammation. COX is known for its inherent property to promote prostaglandin synthesis, resulting in inflammation and pain [7–9]. Therefore, homeostasis of the dental pulp is crucially dependent on the intricate interaction between invading microorganisms and host cells that comprise pulpal soft tissues. The resulting induction of host inflammatory products play a significant role in not only overcoming microbial insults, but also in initiating tooth pain as an early clinical symptom of irreversible pulpitis.

Previous studies have elucidated the contribution of melatonin on the modulation of host tissue homeostasis in a number of tissues. Melatonin is produced from the mammal pineal gland by melanocytes and is known for its distinct versatility on the variety of tissues such as salivary glands and the oral epithelium [10–12]. In bony tissues, administration of melatonin increases mineral components and bone mass, possibly due to an inhibition of receptor activator of nuclear factor kappa-B ligand (RANKL), and induction of osteoprotegerin (OPG), as well as with osteogenic gene expression i.e. bone sialoprotein, osteocalcin, and alkaline phosphatase [13]. It is thus suggested that melatonin is responsible for the maintenance of hard tissue homeostasis.

It is important to note that immunomodulatory properties of melatonin have been extensively investigated. Melatonin potentiates an inflammatory immune response in human myeloid cells involved in both innate and adaptive immunity [14]. Remarkably, previous studies have shown that melatonin is able to regulate glucocorticoid receptor and promote apoptotic resistance in the thymus [15, 16]. In addition, melatonin suppresses LPS-mediated nuclear factor-kappa B (NF- κ B) signaling in human stem cells derived from dental pulp [17], suggesting its antagonistic activity at human TLR4. Collectively, current evidence suggest that melatonin is an essential component required for normal cellular functioning. However, whether melatonin plays a role in LPS antagonism in pulpal fibroblasts during pulpal inflammation still remains poorly understood. Therefore, the objective of this study was to determine an in situ expression of melatonin in human dental pulpal tissues as well as melatonin receptor 1 (MT1) and melatonin receptor 2 (MT2). In addition, a potential melatonin-mediated immunomodulatory mechanisms utilized by pulpal fibroblasts in response to LPS, a major virulent factor of gram-negative microbes, was investigated. Altogether, our in vitro investigations indicate a potential role of pulpal melatonin in fine tuning inflammatory responses to LPS infection.

## Methods

### Preparation of pulpal tissue explants

The need for consent was waived by Khon Kaen University Ethics Committee for human research based on the declaration of Helsinki (Reference number: HE592265). Freshly extracted intact third molars (*n* = 3) without carious lesions from dental patients who underwent a simple extraction procedure were collected immediately in cold sterile Dulbecco’s modified Eagle’s medium (DMEM) (Gibco by Life Technologies, Grand Island, NY, USA) supplemented with 10% heat-inactivated fetal bovine serum (HyClone Laboratories, Logan, UT, USA), 2 mM L-glutamine (Gibco by Life Technologies, Grand Island, NY, USA), 100 U/mL penicillin, and 100 μg/mL streptomycin. Each tooth was washed thoroughly with 1X phosphate buffer saline (PBS) and the periodontal tissue was removed. A carborundum disc was used to cut around the cemento-enamel junction and the cervical portion between crown and root was subsequently split. Pulpal tissue was removed by using sterile forceps and immediately submerged in a 35-mm culture dish containing DMEM. Extirpated pulpal tissue was cut into 1-mm piece using scalpel blade no.15 and was incubated at 37 °C under 5% CO_2_ environment and 95% relative humidity. Culture medium was replenished every 2 days until 100% cell confluence of pulpal fibroblasts was achieved at which the grown cells were passaged.

### Activation of cultured pulpal fibroblasts

Third to sixth passage of isolated pulpal fibroblasts was used in the experiments when cultured cells reached 80% confluence. Stimulation of cultured pulpal fibroblasts with *Escherichia coli* LPS (Sigma-Aldrich, St. Louis, MO, USA) was performed at 20 μg/mL final concentration in growth medium. Dose of LPS stimulation was tested from our preliminary experiments to be effective in the transcript induction of inflammatory mediators. Purified melatonin (Sigma-Aldrich, St. Louis, MO, USA) was used in a wide dose range for stimulation (1 mM, 1 μM, 1 nM, and 1 pM), predetermined for their safety to pulpal fibroblasts by cell viability assays. Cultures of pulpal fibroblasts were stimulated for 3 h or 24 h, as indicated in Figs. 6 and 7, prior to RNA extraction procedures.

### RNA isolation and semi-quantitative RT-PCR analysis

Total RNA from cultured pulpal fibroblasts was extracted and purified using RNeasy mini kit (Qiagen GmbH, Hilden, Germany) according to the manufacturer’s protocol and subsequently tested for its purity. Reverse transcription and polymerase-chain reactions (RT-PCR) were performed separately by using a two-step procedure. First, cDNA was synthesized using High-Capacity cDNA Reverse Transcription Kit (Thermo Fisher Scientific, Carlsbad, CA) with 150 ng of total RNA. Control sample without reverse transcriptase enzyme as well as control without RNA template were prepared similarly and served as negative controls. Next, each PCR reaction was setup using TopTaq Master Mix kit (Qiagen GmbH, Hilden, Germany) to a total volume of 50 μL, containing 15 ng cDNA and 200 nM of each primer sequence. List of primer pairs used in this study was shown in Table 1. Primer sequences used in this study were checked for their specificity using NCBI Primer-BLAST and found that each primer pair shows 100% homology with specific nucleotide sequences of target genes. PCR amplification were subsequently carried out by following the optimal conditions for each primer-dependent PCR reaction comprised of denaturation, annealing, and extension for 40 cycles. Glyceraldehyde-3-phosphate dehydrogenase (GAPDH) was used as housekeeping gene control. After each run, gel electrophoresis was performed with 20 μL PCR product combined with 1X loading dye in 2% agarose gel, and visualized under UV light to examine the specificity of PCR products. No spurious product was detected from each amplification. Densitometric analysis of the intensities of PCR products relative to GAPDH control was performed by imaging an agarose gel using a gel documentation system (Syngene, Cambridge, UK). An average mRNA expression level was subsequently calculated from 3 different donors whose healthy pulpal fibroblasts were isolated and was presented as mean ± standard deviation.

**Table 1:** List of primers used in the study

Gene	Primer sequence (5′➔ 3′)	Predicted product size (bp)	Reference
GAPDH	Forward: ATGACCCCTTCATTGACCTCA Reverse: GAGATGATGACCCTTTTGGCT	265	Shukla S et al. Int J Oncol. 2009
COX-1	Forward: GAGTCTTTCTCCAACGTGAGC Reverse: ACCTGGTACTTGAGTTTCCCA	350	Choi EK et al. FEBS Lett. 2004
COX-2	Forward: GCAGTTGTTCCAGACAAGCA Reverse: CAGGATACAGCTCCACAGCA	359	Fernau NS et al. J Biol Chem. 2010
IL-1 β	Forward: CCAGGGACAGGATATGGAGCA Reverse: TTCAACACGCAGGACAGGTACAG	129	Abe A et al. Pathol Int. 2014.
MT1	Forward: GATCCTGGTTGTCCAGGTCA Reverse: CATTGAGGCAGCTGTTGAAA	241	Tachibana R et al. Int J Mol Sci. 2014.
MT2	Forward: CAACTGCTGCGAGGCG Reverse: GGCGGTGGTGACGATG	133	Lanoix D et al. Hum Reprod. 2006.

### Specimen preparation for immunohistochemistry

Pulpal tissues from freshly extracted third molars, 12 sound third molars and 6 third molars diagnosed with irreversible pulpitis without apical periodontitis or abscess, were collected by splitting off the roots and immediately placed the pulpal tissues in 10% formalin for 24 h prior to paraffin embedding, similar to a previously published study [18]. Embedded pulp specimens were then cut into 5-μm thickness and collected onto coated glass slides for further analysis.

Immunohistochemistry analysis was performed in paraffin-embedded pulpal tissues. Briefly, Peroxo-Block™ (Invitrogen, Paisley, UK) was used to inhibit endogenous peroxidase activity. Specimens were subsequently blocked for non-specific antibody binding with Protein Block Serum-free (DAKO, Carpinteria, CA, USA). Immunohistochemistry staining was performed by using antibodies raised against human proteins as follows: rabbit anti-human melatonin (5 μg/mL; Thermo Scientific, Rock ford, IL, USA), rabbit anti-human MT1 (25 μg/mL; Abnova, Taipei, Taiwan), rabbit anti-human MT2 (50 μg/mL; Abnova, Taipei, Taiwan). Concentrations of primary antibodies used in the study were optimized for their optimal immunostaining signals in our preliminary experiment. Each primary antibody were incubated with tissue sections separately for 24 h at 4 °C overnight. Tissue slides were thoroughly washed with PBS supplemented with 0.5% Tween (PBS-T) and incubated with anti-rabbit secondary antibody (DAKO EnVision^+^ System-HRP labeled polymer, Carpinteria, CA, USA). Diaminobenzidine detection system was employed by the addition of DAB tetrahydrochloride solution (DAKO Liquid DAB^+^ Substrate Chromogen System, Carpinteria, CA, USA) onto HRP-conjugated tissue sections. Sections were then counterstained with hematoxylin (Mayer’s hematoxylin, Bio Optica, Milano, Italy), dehydrated and mounted. Tissue biopsy specimens from human pineal glands were stained similarly and used as a positive control, while omission of primary antibodies served as a negative control.

### Immunohistochemistry analysis of melatonin, MT1, and MT2 expression in pulpal tissues

Sectioned pulpal tissues stained for human melatonin from 5 healthy third molars were investigated by using a light microscope (Nikon ECLIPSE 80i, Nikon Singapore Pte. Ltd., Mapletree Anson, Singapore) under 10X eyepiece lens coupled with 20X objective lens for the staining profile of melatonin. Descriptive analysis was used to qualitatively evaluate the signal intensities emanated from each layer of pulpal tissues. For MT1 and MT2 analysis, approximation of stained cells was enumerated from 3 tissue sections obtained from each pulp sample. Total number of 12 healthy pulps and 6 inflamed pulps were fixed and stained for human MT1 and MT2. Each slide was blindly scored for the percentage of stained cells under a light microscope at 200X magnification. Semi-quantitative rating system was utilized to assess the distribution of stained cells and number of positively stained cells. Level of MT1 and MT2 expression within pulpal tissues was scored according to the relative intensity of the staining signals on a scale of 0 to 4 (0 = no positively stained cells, 1 = amount of positively stained cells less than 25%, 2 = amount of positively stained cells between 25 and 50%, 3 = amount of positively stained cells between 50 and 75%, and 4 = amount of positively stained cells more than 75%). Histological assessment and rating of staining intensity were performed twice more time with one-week time gap to minimize recall bias.

### Statistical analysis

**Fig. 1:**
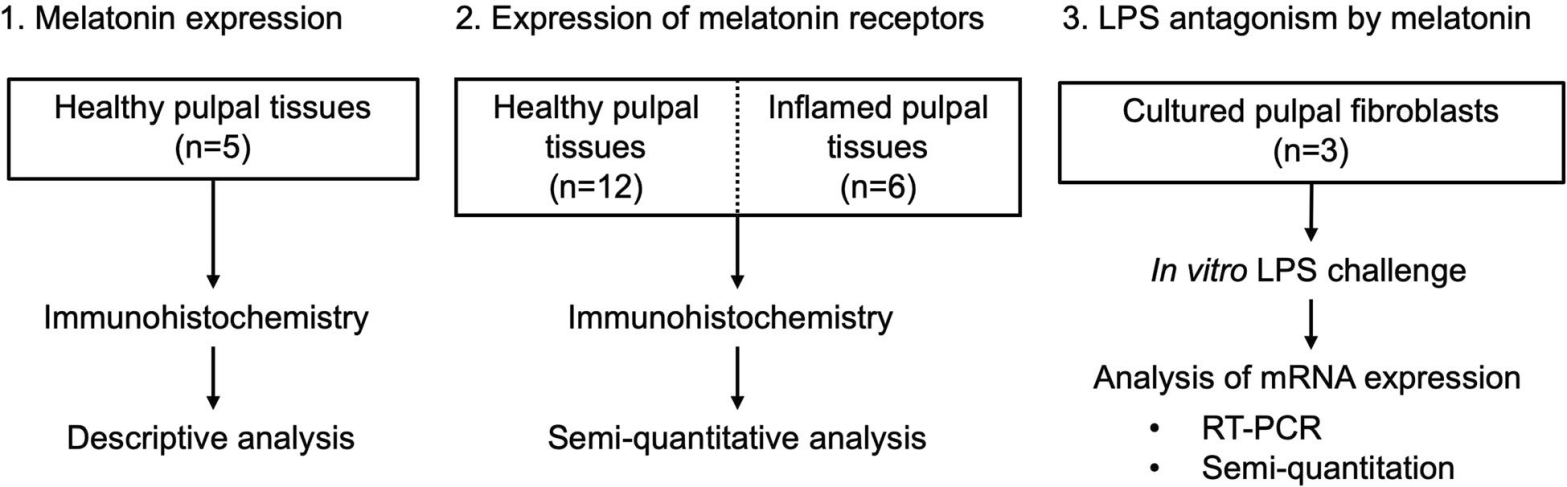
A flow chart summarizing the experimental procedures incorporated in this study

All experiments conducted in this study were summarized in Fig. 1. All presented data were derived from 3 independent experiments yielding identical results. In experiments using human tissue samples, a minimum of 3 specimens harvested from different donors were tested in triplicate determinations. Relative staining intensities of MT1 and MT2 were compared between healthy and inflamed pulps by using Mann-Whitney U test. Level of mRNA expressions was compared by using One-way ANOVA. Data analysis was performed by using a GraphPad Prism version 7 (GraphPad Software Inc., La Jolla, CA, USA). Calculated *P*-value of less than 0.05 was considered statistically different.

## Results

### Abundance of melatonin expressed in pulpal tissues

**Fig. 2:**
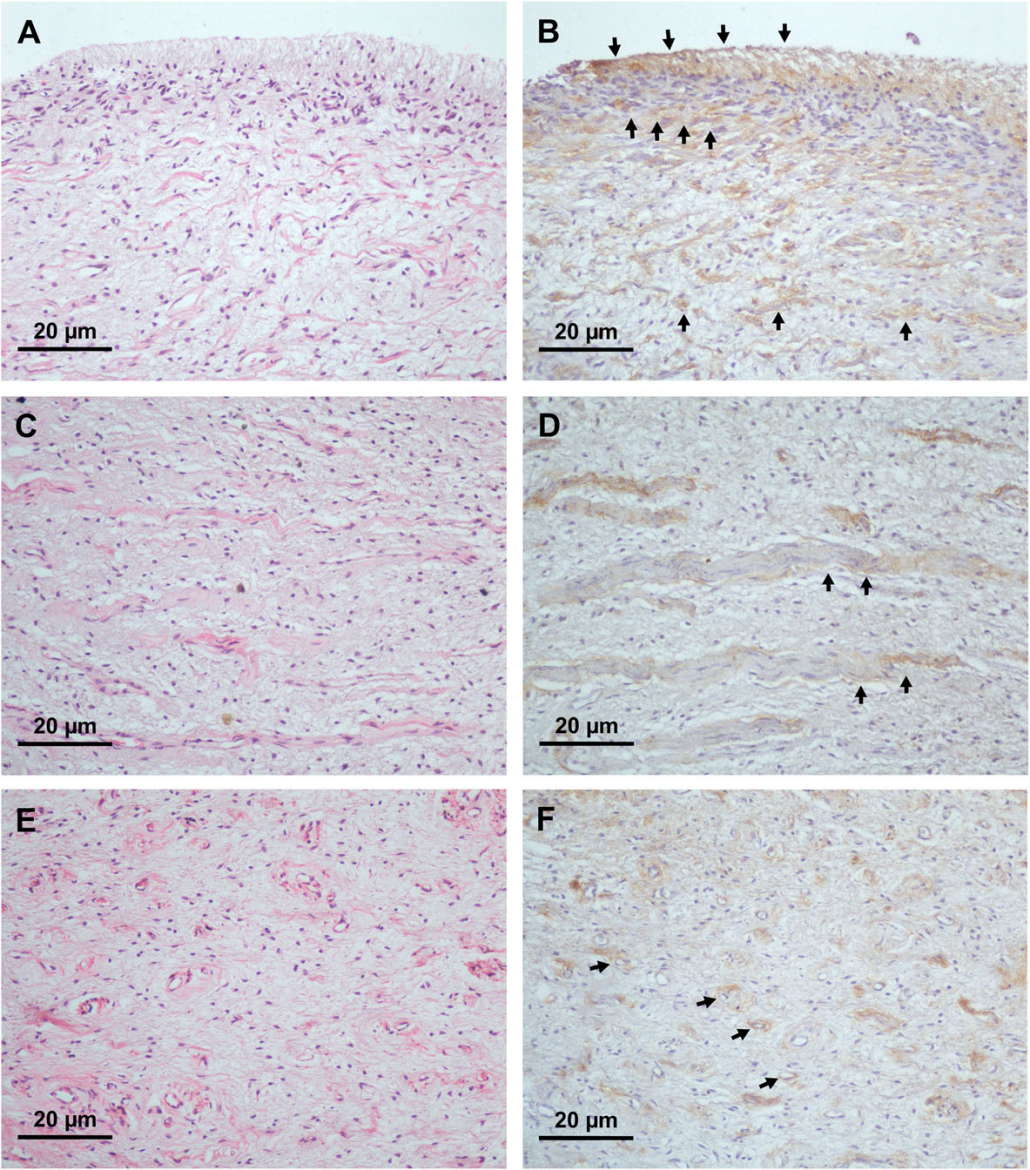
Histological staining against human melatonin expressed in pulpal tissues. 2A, 2C, and 2E illustrate hematoxylin and eosin staining (H&E) of normal pulp at 200X magnification level. A strong staining signal was observed in odontoblastic layers and pulpal connective tissues (arrows; 2B). Localization of melatonin with collagen-like substances (arrows; 2D) and endothelial cells (arrows; 2F) was evident in normal pulp

We first sought to determine whether melatonin protein was expressed in dental pulp. Histological structure of normal pulpal anatomy was shown by H&E staining (Fig. 2a, c, and e). Immunohistochemistry staining of in situ melatonin expression in dental pulpal tissues showed that melatonin was strongly localized in the superficial layer of pulpal tissues, particularly in the cell-rich zone and odontoblastic layer (Fig. 2b). Localization of melatonin was also detected in the connective tissue layer, densely packed with collagenous substances (Fig. 2d). It is important to note that melatonin was also colocalized with endothelial cells (Fig. 2f). Pattern of expression suggests that melatonin is a constitutive protein expressed in healthy dental pulps, and its magnitude of expression is increased toward the dentin-pulp interface.

### Contrasting expression of melatonin receptors

**Fig. 3:**
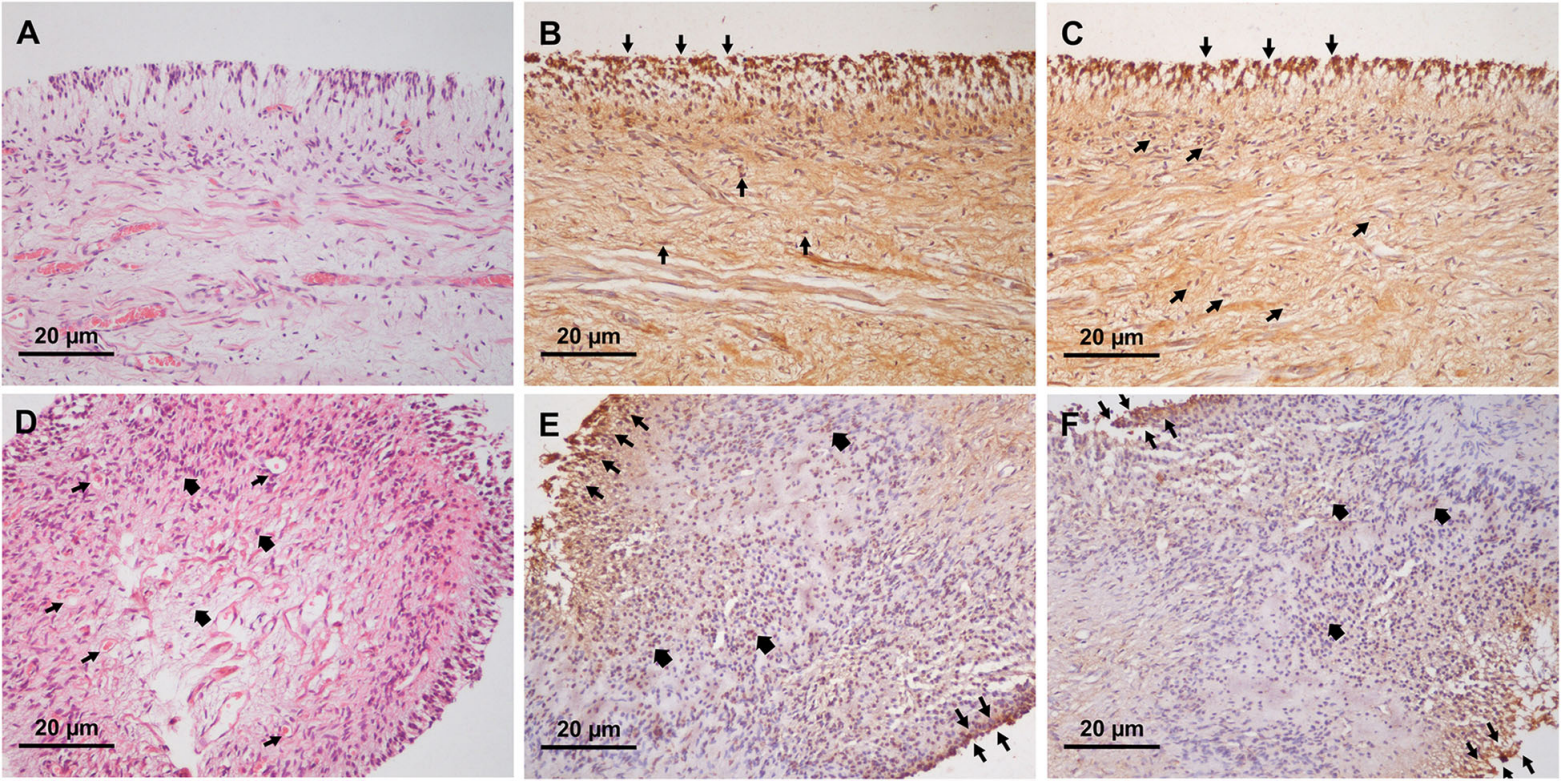
Expression of melatonin receptors MT1 and MT2 in normal and inflamed pulps. 3A and 3D show H&E staining of normal and inflamed pulp under 200X magnification level, respectively. 3B and 3C illustrate MT1 and MT2 staining in normal pulps, whereas 3E and 3F show MT1 and MT2 expression profiles in inflamed pulp. In normal healthy pulps, both MT1 and MT2 were intensely expressed in odontoblastic layer and cell-rich zones. Co-localization of MT1 and MT2 with pulpal fibroblasts was observed in pulpal stroma (arrow heads; 3B and 3C). In inflamed pulp, increased vascularization (arrows; 3D) and leukocyte infiltration (bold arrows; 3D) were seen in pulpal stroma. Similar to normal healthy pulps, MT1 and MT2 were found in odontoblastic layers (arrows; 3E and 3F) and co-localized with infiltrating leukocytes located in the pulp core (bold arrows; 3E and 3F)

**Fig. 4:**
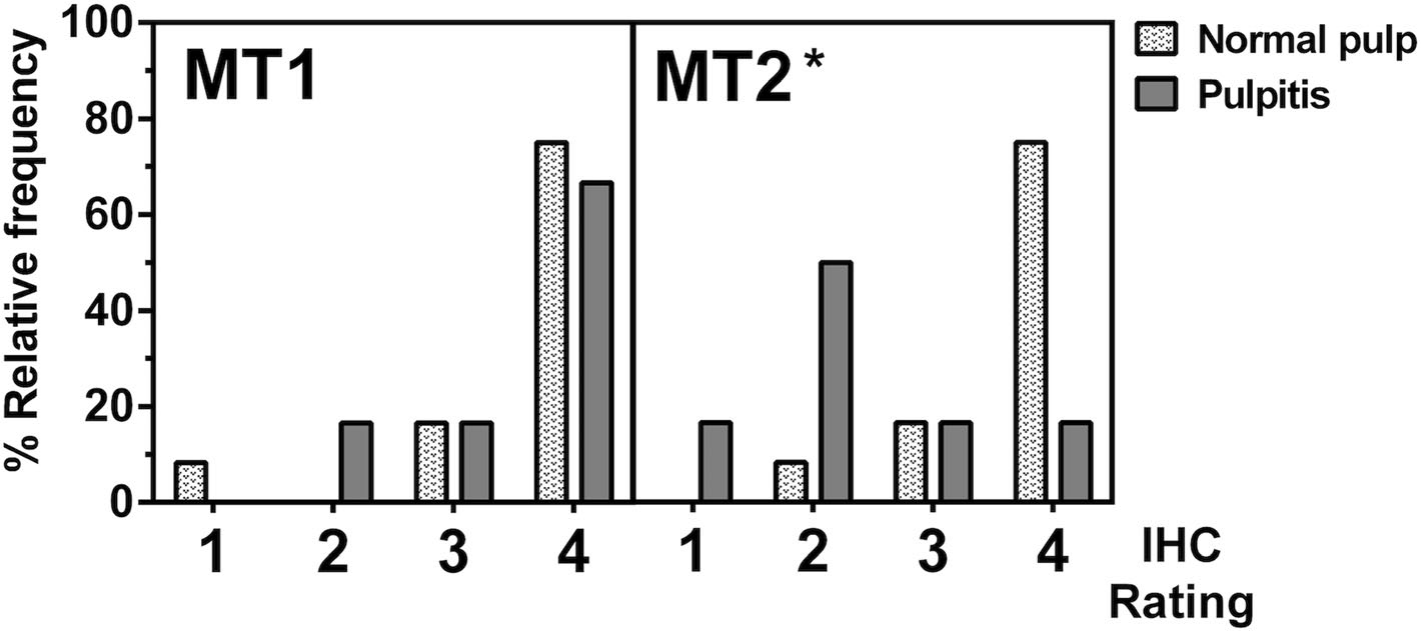
Histogram shows a relative frequency of MT1 and MT2 signal intensities from an immunostaining of paraffin-embedded normal and diseased pulps. A scale of 0–4 indicates the level of expression from the weakest to the strongest staining signals. Please note that none of the stained samples was scored as 0. Statistical analysis found that MT2 expression in normal pulps was significantly different from that of inflamed pulps (*P*-value< 0.001), whereas MT1 expression compared between health and disease of the pulp was not statistically different (*P*-value = 0.559). Asterisk (*) indicates statistical difference at *P*-value < 0.05 as determined by using Mann-Whitney U test

Next, we investigated the expression of MT1 and MT2 in pulpal tissues in relation to the health of the pulp. Our H&E staining demonstrated that, in healthy pulps, dental pulp periphery was comprised of an intact palisade of odontoblast linings (Fig. 3a), while disintegration of an odontoblastic layer was observed in inflamed pulps (Fig. 3d; top right corner). Our histological analysis revealed that, in healthy pulps, both MT1 and MT2 proteins were detected predominantly in odontoblastic zone, cell-rich zone, and pulp core. The strongest staining signal was from the odontoblastic cell layer (Fig. 3b and c) and the majority of tissue sections showed that approximate number of positively-stained cells was more than 75% under 200X magnification level. In diseased pulp, increased numbers of infiltrating leukocytes (bold arrows, Fig. 3d) were seen as well as an increase of capillaries in the pulpal stroma (arrows, Fig. 3d). Notably, expression of MT1 was found predominantly in odontoblastic layer (arrows, Fig. 3e) and co-localized with infiltrating leukocytes located in pulpal stroma (bold arrows, Fig. 3e). The majority of stained pulpal tissues obtained from diseased pulp was scored as 4, with considerable numbers of cells more than 75% positively stained as indicated by brown nuclear staining (Fig. 3e). However, MT2 expression dramatically decreased, with a significant number of samples showing staining intensity graded to a level 2 (Fig. 3f). Mann-Whitney U test further verified that the relative staining signal of MT2 was statistically different (*P*-value< 0.001) between normal and inflamed dental pulpal tissue (Fig. 4). MT1 expression in disease showed no statistical difference (*P*-value = 0.559) compared to its expression in health. Our results demonstrated a striking contrast between MT2 and MT1 expression, with a significant decrease in MT2, but not MT1, in inflamed pulpal tissues.

### Melatonin antagonized LPS-induced signaling of inflammatory mediators in pulpal fibroblasts

**Fig. 5:**
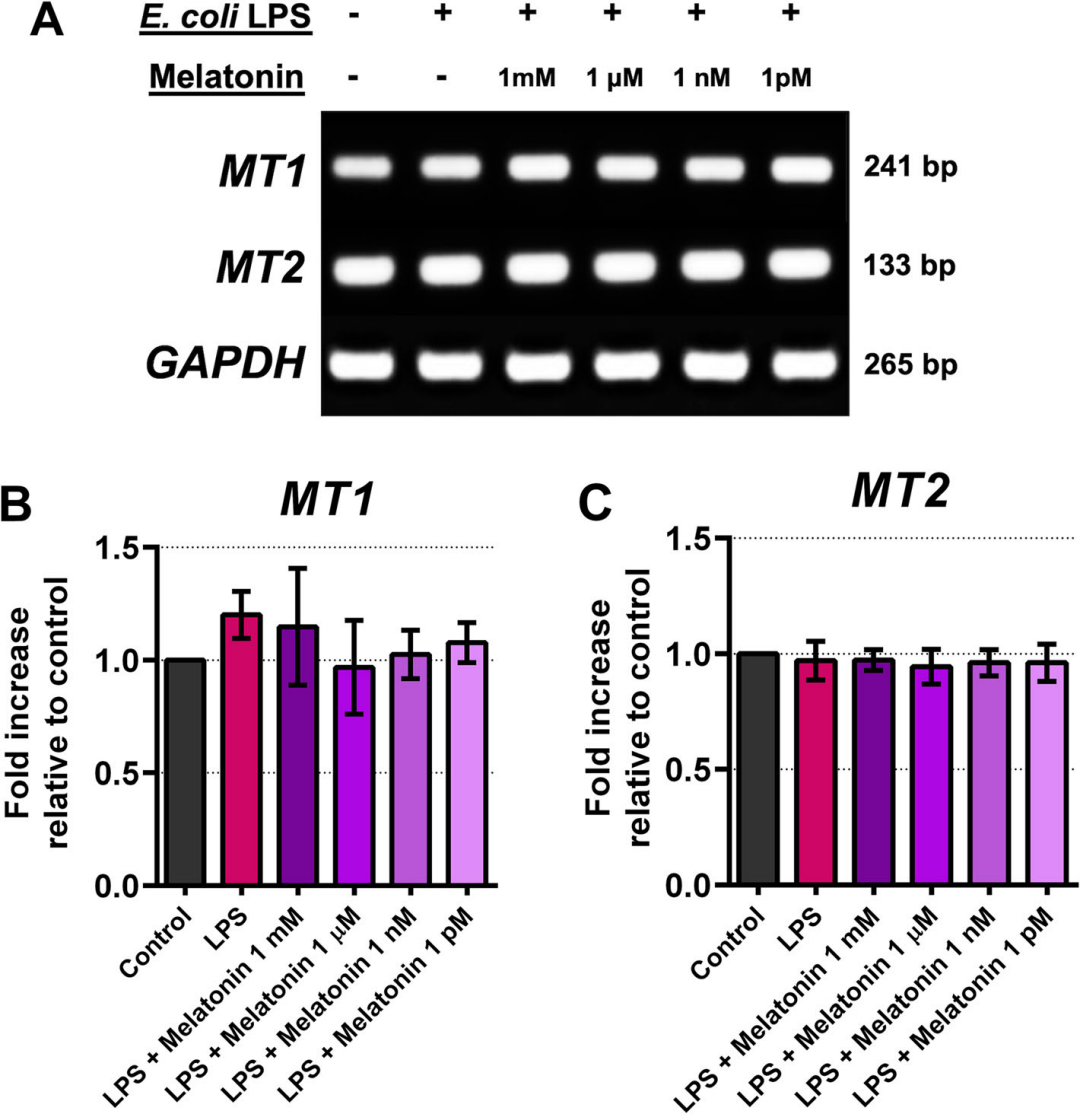
Expression of *MT1* and *MT2* transcripts of cultured pulpal fibroblasts after co-incubation with *E. coli* LPS (20 μ g/mL) and exogenous melatonin at different concentration for 24 h. Relative densities of the representative PCR products from one of 3 independent experiments yielding similar results were shown (5A), in which one-way ANOVA did not reveal any statistical difference (5B, and 5C)

**Fig. 6:**
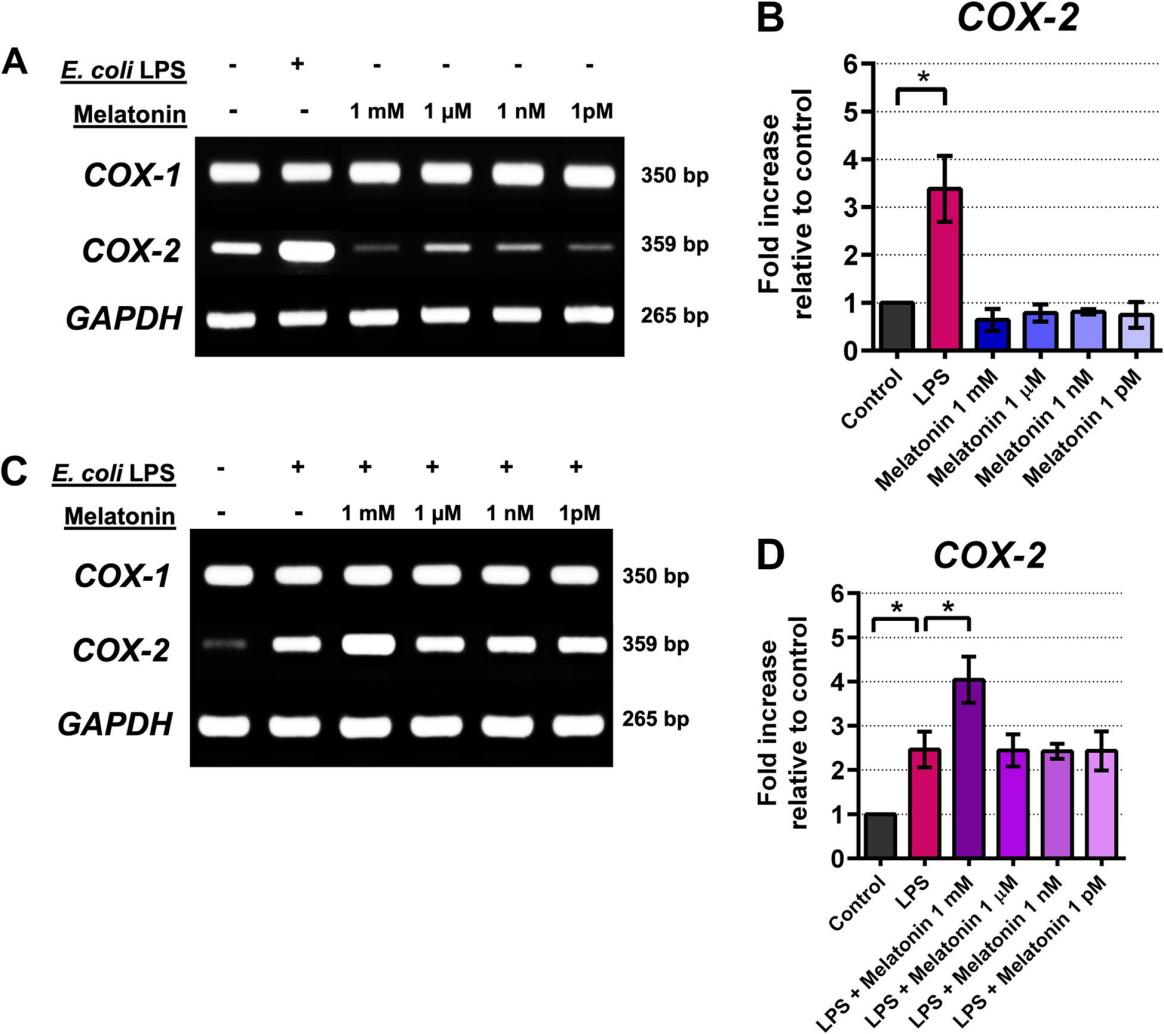
Expression of *COX-1* and *COX-2* transcripts of cultured pulpal fibroblasts after incubation with *E. coli* LPS (20 μ g/mL) and/or various concentrations of exogenous melatonin. After 3 h of incubation with *E. coli* LPS, while *COX-1* mRNA expression was remained unaffected, *COX-2* was increased when incubated with LPS alone (6A). Relative densities of the bands from one of 3 independent experiments yielding similar results were shown (6A, 6C). Addition of exogenous melatonin suppressed basal *COX-2* expression (6A, 6B). Loss of LPS antagonism by melatonin after 24 h of incubation was observed (6C, 6D). Asterisk (*) indicates statistical difference at *P*-value < 0.05 as determined by One-way ANOVA with Bonferroni’s multiple comparisons

**Fig. 7:**
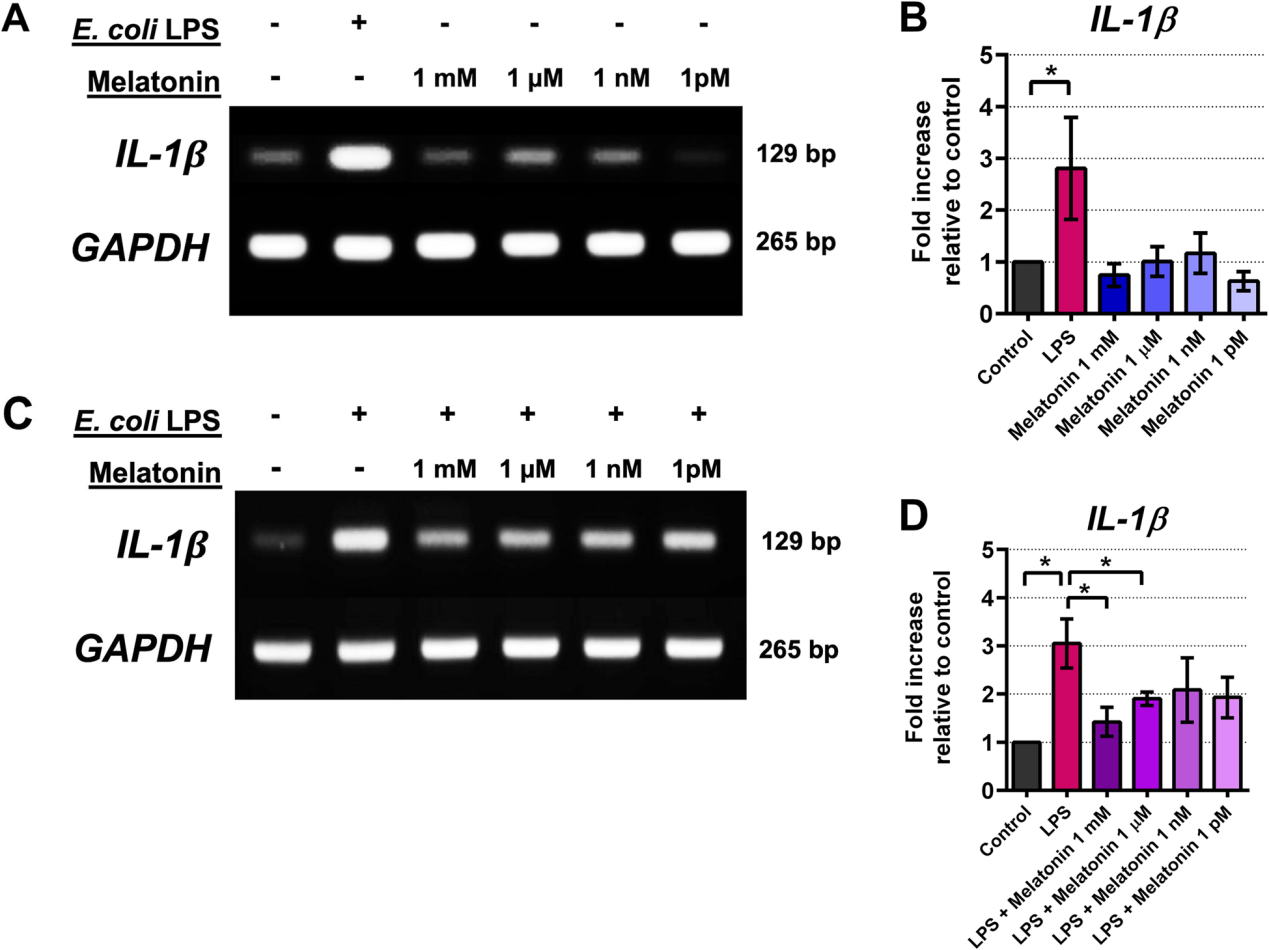
Expression of *IL- β* transcripts of cultured pulpal fibroblasts after incubation with *E. coli* LPS (20 μ g/mL) and/or various concentrations of exogenous melatonin. Relative densities of the bands from one of 3 independent experiments yielding identical results were shown (7A, 7C). Basal expression of *IL- β* transcripts was not affected by melatonin co-cultured with pulpal fibroblasts for 3 h (7A, 7B). Co-culture of LPS with melatonin reduced of LPS-induced *IL- β* expression after 24 h of incubation, particularly 1 mM and 1 μ M melatonin (7C, 7D). Asterisk (*) indicates statistical difference at *P*-value < 0.05 as determined by One-way ANOVA with Bonferroni’s multiple comparisons

The involvement of melatonin in antagonism against pulpal inflammation was investigated in explanted and cultured human pulpal fibroblasts, specifically examining the potential modulation of COX and IL-1 β expression. First, we examined whether transcripts of melatonin receptors, MT1 and MT2, were expressed in cultured pulpal fibroblasts. Results show that after 24 h all test cell lines expressed robust *MT1* and *MT2* mRNAs (Fig. 5a, b, and c). Levels of mRNA expression of these melatonin receptors were not statistically different as shown by semi-quantitative mRNA analysis (Fig. 5b and c). This suggests there may be a prompt engagement of pulpal fibroblasts in response to exogenous melatonin upon the activation of melatonin receptors. Secondly, when pulpal fibroblasts were infected with 20 μ g/mL *E. coli* LPS, there was an increased expression of *COX-2*, indicating that one of the intracellular signalings is mediated through the COX-2 pathway (Fig. 6a). Surprisingly, while *COX-2* mRNA was upregulated by the addition of LPS, strong basal *COX-1* expression was seen and remained unaffected when either LPS or melatonin was added (Fig. 6a and c, S1, and S2). Moreover, addition of melatonin for 3 h significantly suppressed basal *COX-2* mRNA expression (Fig. 6a), although not statistically significant (Fig. 6b). Our densitometric analysis revealed that upon the addition of melatonin into culture, *COX-2* transcript was significantly reduced relative to basal *COX-2* expression, with maximal reduction when 1 mM melatonin was added (Fig. 6 and b). Thus, this indicates that melatonin may play an antagonistic role against COX-2 induction in pulpal fibroblasts. We also found similar findings when *IL-1 β* transcript was examined (Fig. 7a), showing that LPS significantly upregulated *IL-1 β* as determined by One-way ANOVA (Fig. 7b). Our mRNA data thus suggest that melatonin targets multiple signaling pathways to dampen an inflammatory response by pulpal fibroblasts. Surprisingly, LPS antagonism by melatonin via COX-2 signaling was not as pronounced (Fig. 6c and d) when compared to IL-1 β after co-cultured for 24 h (Fig. 7c and d). It is also striking that *COX-2* was up-regulated in the presence of 1 mM melatonin in vitro, and none of other melatonin concentrations was able to suppress *COX-2* expression induced by LPS at 24 h after infection (Fig. 6c and d). This might suggest an antagonistic effect of melatonin on COX-2 signaling under an optimal culture condition. In contrast, *IL-1 β* expression was down-regulated in a dose-dependent manner, particularly when pulpal fibroblasts were co-cultured with LPS and 1 mM melatonin (Fig. 7c and d). Taken together, our findings highlight that melatonin has selective antagonistic characteristics for LPS-mediated inflammatory signal induction via COX-2 and IL-1 β.

## Discussion

Immune response of dental pulp to microbial stimuli is triggered via a repertoire of innate immune sensors, such as TLRs including LPS sensor TLR4. Concurrently, host proteins released in response to microbial recognition loop back to autoregulate and fine tune host defense response. For one example, TGF- β 1 released after TLR4 engagement, autoregulates TLR4 signaling in odontoblasts by suppressing TLR4 at both mRNA and protein levels, dampening the inflammatory response [5]. Similarly, melatonin is able to modulate immune responses in pulpal stem cells [17]. Worth noting is that melatonin is globally expressed in different tissues and possibly affects their phenotypes. Notably, expression of melatonin was found to be significantly upregulated in oral lichen planus lesions. Morphometric analysis of oral mucosa in oral lichen planus lesions showed an abundance of MT1 in the spinous cell layer and connective tissue layer of the epithelium when compared to normal epithelium [18]. This expression pattern suggests the association of melatonin and its receptor with the pathogenesis of oral mucosal disease. Remarkably, melatonin and its derivatives exert anti-oxidative effects against human gingival fibroblast cell death induced by hydrogen peroxide possibly via several mechanisms including the inhibition of nitric oxide synthesis and reduced lipid peroxidation [19]. Melatonin was also detected in saliva and gingival crevicular fluids, and the amount was surprisingly higher in subjects with healthy periodontium compared to individuals with periodontitis [20, 21]. It is thus possible that an increased expression of melatonin in the oral epithelium provides an instant cytoprotective barrier against immune-mediated cellular damage of oral epithelial cells [22].

In healthy pulpal tissues, the strongest melatonin expression was in odontoblastic layers consisting of the cell-rich zone. In addition, abundant melatonin was found in pulpal stromal cells and in endothelial cells. This expression profile is analogous to the transepithelial gradient of IL-8, where IL-8 was found most abundantly on the superficial epithelial cell layer in juxtaposition to subgingival plaque [23]. Even in caries-free teeth, melatonin-expressing cell layers in dental pulp may thus act as instant front-line defenders in response to an approaching microbial burden. There it is postulated that melatonin in dental pulp may be inducible by a cariogenic microbiome and requires further investigation.

This study also showed that odontoblasts and pulp cells express melatonin receptors MT1 and MT2. Notably, an elevated expression level of MT1 but not MT2 was correlated with diseased pulp. In the developing human tooth, MT1 was found to be expressed in odontoblasts and dental papilla cells [24]. Our immunohistochemistry data have led to the hypothesis that MT1 plays a pivotal role in the synthesis of reactionary dentin in response to polymicrobial infection in the pulp due to an increased MT1 expression during pulpal inflammation. Contrasting expression profiles of melatonin receptors MT1 and MT2 during pulpal disease suggest that coordination between these two receptor isoforms may play an essential role in the modulation of innate immunity within the pulp, and that these two receptors might have an opposite function in the pulp. MT1 may presumably act as a selective sensor to recognize melatonin synthesized in response to microbial infection. In agreement with this notion, increased MT1 expression was found in osteosarcoma cells, relative to normal bone cells [25], thus indicating the responsiveness of MT1 in bony pathogenesis. However in contrast to MT1, there is limited knowledge about the function of MT2. It has been shown that stimulation of melatonin receptors with their specific agonist Ramelteon increased survival in rats with polymicrobial sepsis [26]. In line with this finding, administration of short-term melatonin in septic mice reduced rates of septic shock compared to mice treated with a vehicle control [27]. Topical application of melatonin on oral mucosal surfaces has been shown to reduce RANKL, but increase OPG levels in saliva, suggesting the efficacy of melatonin on arresting the destruction of periodontal tissue [28]. Altogether, these studies suggest that melatonin receptors may play protective roles when activated by melatonin. Therefore, it is possible that during microbial infection-induced pulpal inflammation, increased melatonin inhibits pulpal inflammation via local engagement with melatonin receptors abundantly expressed in inflamed pulps.

Similar to other tissue sites, pulpal inflammation is associated with an intricate interaction between host inflammatory mediators and host cells under a hostile microenvironment of various inflammatory mediators. Induction of COX enzymes is a prerequisite for prostaglandin synthesis, particularly COX-2 that is known for its inducible profile in response to bacterial LPS and inflammatory cytokines [29–31]. In inflamed dental pulps, COX-2 expression was found predominantly in the area of infiltrating leukocytes, but was rarely detectable in normal healthy pulps [32]. Importantly, the presence of pleiotropic inflammatory cytokines such as IL-1 α and TNF- α in addition to microbial stimuli in the microenvironment induced a robust COX-2 response in cultured pulp cells [33]. These findings highlight a significant role of COX-2 signaling in pulpal innate immune defense, particularly by pulpal fibroblasts. Black-pigmented pathogenic bacteria including *Porphyromonas endodontalis* also conferred the ability to induce COX-2 expression in human pulp cells [34]. Our in vitro investigation in cultured human pulpal fibroblasts harvested from dental pulp tissue explants showed that COX-2, but not COX-1, was induced by *E. coli* LPS after stimulation for 3 h. Importantly, COX-1 was constitutively expressed in dental pulp cells harvested from 3 donors which had a negligible increase of COX-1 transcript when stimulated with LPS.

Surprisingly, we found that the addition of exogenous melatonin into the culture medium attenuated basal expression of COX-2. This finding underscores the requirement of a select signaling pathway for pulpal homeostasis via COX-2 induction in dental pulp. Co-culture of LPS-stimulated pulpal fibroblasts with exogenous melatonin reduced COX-2, but not COX-1, during the early-phase response to LPS infection. This antagonistic phenotype was lost during late phase of LPS stimulation. Loss of LPS antagonism mediated by melatonin after 24-h may be the result of autocrine activity of inflammatory cytokines such as IL-1 β and TNF- α that automatically induce COX-2 expression. It is possible that COX-2 signaling is necessary for fine tuning an adaptive immune response within the pulp providing that prostaglandin E2 (PGE2) is able to promote vascular permeability [35], and induces immunoreactive calcitonin gene-related peptide (iCGRP) via bradykinin, a mechanism believed to be responsible for pulpal pain [36]. Interestingly, COX-2 deletion resulted in a decreased level of IL-1 β and interferon- γ in response to LPS infection in vivo [37]. Thus, in the context of the pulpal environment, inhibition of inflammatory cascades via LPS-induced COX-2 upregulation may result in a modified adaptive inflammatory response through the modulation of PGE2 synthesis and suppression of COX-2, while simultaneously reducing peripheral sensitization. Whether LPS-stimulated pulpal fibroblasts utilize this mechanism to maintain tissue homeostasis requires additional examinations.

A recent study has shown that systemic administration of melatonin during LPS-induced pulpal inflammation dampened TLR4-mediated inflammatory response in dental pulp cells [17]. Our results were consistent with their findings as a 3-h co-culture of *E. coli* LPS-stimulated human pulpal fibroblasts with melatonin reduced *COX-2* and *IL-1 β* transcripts. Antagonism mediated by melatonin is thought to occur in part at TLR4 because LPS derived from *E. coli* exerts host hyperresponsiveness specifically at human TLR4 [38, 39]. Although not examined in this study, previous investigations have demonstrated the abundance of TLR4 in human pulpal fibroblasts [2, 40] and LPS infection increased PGE2 and COX-2 expression [40], indicating the participation of TLR4 in pulpal innate immune response during an infection with cariogenic microbes. Remarkably, LPS-induced human dental pulp stem cells (DPSCs) upregulated the expression of functional TLR4 and increased cell migration, but resulted in a reduced proliferation of DPSCs [41]. This suggests there is a distinct local immune response to bacterial stimuli by the recruitment of dental pulp cells in deep dental caries. Interestingly, concomitant activation of eukaryotic cells with bacterial LPS and melatonin seemingly modulates an expression of inflammatory mediators. An earlier study has demonstrated that melatonin suppressed LPS-induced acetylation of the p52 isoform of NF- κ B, an important activation step for COX-2 induction in macrophages [42]. In addition, melatonin blocked the activation of NLRP3 inflammasome required for IL-1 β biosynthesis [43]. In agreement with these notions, we clearly elucidated the antagonistic activity of exogenous melatonin against LPS infection of pulpal fibroblasts via the suppression of COX-2 and IL-1 β. It is striking that melatonin similarly suppressed TLR4 and NF- κ B in mice infected with LPS and provided protection against mastitis [44]. However, whether pulpal fibroblasts utilize blocking mechanisms at NLRP3 and the p52 NF- κ B subunit to regulate chronic pulpal stimulation remains to be elucidated. Collectively, our results might shed light on a functional interplay between melatonin receptors and TLR4 in pulpal fibroblasts. Activation of innate immune receptors in dental pulp by invading bacteria along with pulpal hormone signaling may coordinate host receptors with distinct functions to enhance dental pulp defense. However, the influence of physiological conditions of the body on pulpal melatonin level is still unclear. Whether pulpal melatonin is induced in response to polymicrobial infection found in dental caries also warrants additional investigation. Considering the inhibition of cellular activities by melatonin, multiple intracellular signaling cascades could be targeted by exogenous melatonin deposited in extra-pineal tissues and may be a suitable candidate hormone that could be used in targeted therapy of inflammatory diseases.

## Conclusion

In summary, we have reported the ubiquity of melatonin and its receptors i.e. MT1 and MT2 in human dental pulp and a possible implication of melatonin in pulpal tissue homeostasis via the antagonism of LPS-induced COX-2 and IL-1 β signaling. Abundance of extra-pineal melatonin and its hormone receptors MT1 and MT2 in pulpal tissue suggests that melatonin might license dental pulp an immune regulation both via a direct melatonin-receptor engagement and its cross interactions with pulpal microbial sensors in the vicinity, which has yet to be elucidated. Interesting to note is the increased MT1, but not MT2 expression in inflamed pulps, indicating a distinct utilization of melatonin receptors during pulpal pathogenesis. Melatonin in orchestration with its receptors might thus provide a first line defense against microbial insult in dental pulp. Perhaps, melatonin may prove beneficial for the treatment of inflamed dental pulpal tissues. Nonetheless, induction of melatonin expression by polymicrobial infection especially in pulpal disease still needs further elucidation.

## Supplementary information

**Additional file 1 Fig. S1** Semi-quantitative analysis of *COX-1* mRNA expression by pulpal fibroblasts cultured with LPS or various dose of exogenous melatonin alone for 3 h.

**Additional file 2 Fig. S2** Semi-quantitative analysis of *COX-1* mRNA expression by pulpal fibroblasts stimulated with LPS alone or LPS with the presence of exogenous melatonin in the culture for 24 h.

## Data Availability

All data generated or analyzed during this study are included in this published article (and its supplementary information files).

## Abbreviations

CCL: Chemokine ligand
COX: Cyclo-oxygenase
IL-1 β: Interleukin-1 β
LPS: Lipopolysaccharide
MT1: Melatonin receptor 1
MT2: Melatonin receptor 2
NF- κ B: Nuclear factor-kappa B
TLR: Toll-like receptor
TNF- α: Tumor necrosis factor- α
